# DANCR promotes septic cardiomyopathy by enhancing macrophage glycolytic reprogramming via the IGF2BP2/HK2 axis

**DOI:** 10.3389/fcell.2025.1628915

**Published:** 2025-11-27

**Authors:** Chunhui Ma, Shaoyu Liu, Yu Zhao, Hong Liu, Xuguang Zhang

**Affiliations:** 1 Department of Clinical Laboratory, Affiliated Hospital of Shandong Second Medical University, Weifang, Shandong, China; 2 School of Medical Laboratory, Shandong Second Medical University, Weifang, Shandong, China

**Keywords:** septic cardiomyopathy, DANCR, macrophage M1 polarization, glycolysis, IGF2BP2, HK2

## Abstract

**Background:**

Metabolic reprogramming of macrophages shapes their phenotypic plasticity and contributes to the progression of septic cardiomyopathy. This study investigated the role of DANCR in modulating of macrophage glycolysis, thereby elucidating its mechanism for augmenting septic cardiomyopathy.

**Method:**

THP-1-derived macrophages were stimulated with LPS and subjected to DANCR knockdown or HK2 overexpression. Inflammatory cytokine levels, M1 polarization, and glycolytic activity were evaluated. Conditioned media from treated macrophages were used to treat cardiomyocytes. Mechanistic analyses, including bioinformatics, RNA immunoprecipitation (RIP), and RNA stability assays, were performed to identify downstream targets of DANCR.

**Result:**

DANCR silencing attenuated M1 polarization and activity in macrophages, improved cardiomyocyte viability and reducing apoptosis. Mechanistically, DANCR upregulated HK2 expression by enhancing IGF2BP2-mediated mRNA stabilization. HK2 overexpression reversed the protective effects of DANCR knockdown, whereas pharmacologic inhibition of glycolysis counteracted the effects of HK2 overexpression, confirming the involvement of the DANCR/IGF2BP2/HK2 axis.

**Conclusion:**

DANCR promotes septic cardiomyopathy by enhancing macrophage glycolytic reprogramming via the IGF2BP2/HK2 axis. Targeting this pathway may provide a novel therapeutic strategy.

## 1 Introduction

Septic cardiomyopathy is a reversible structural and functional impairment of the myocardium that occurs as a complication of sepsis. It affects approximately half of patients experience septic with septic shock and is associated with a markedly high mortality rate, ranging from 70% to 90% (Lima and Silva, 2023). Despite its clinical significance, no targeted therapies have been approved specifically for septic cardiomyopathy. Current management strategies focus primarily on standard sepsis treatment protocols and supportive cardiovascular care (Boissier and Aissaoui, 2022). Agents such as norepinephrine are commonly used as first-line vasopressors to maintain mean arterial pressure, and dobutamine is employed to enhance myocardial contractility in cases of cardiac dysfunction (Lukić et al., 2024). However, these agents are primarily symptomatic treatments and do not address the underlying inflammatory or metabolic changes in septic cardiomyopathy. Recently, pharmacologic strategies such as β-blockers (e.g., esmolol) have been explored to modulate heart rate and optimize myocardial oxygen balance, while levosimendan, a calcium sensitizer, has been evaluated for its combined inotropic and vasodilatory effects. However, clinical trials have yielded inconsistent outcomes, and concerns about hypotension or tachyarrhythmias limit their clinical application (Lukić et al., 2024). Despite extensive preclinical and clinical investigation into this condition over the past 3 decades, the pathogenesis of septic cardiomyopathy remains elusive (Wang et al., 2022). Thus, further exploration into the etiologies of septic cardiomyopathy is warranted to optimize intervention strategies and enhance clinical outcomes.

Recent research has increasingly focused on immune cells, particularly macrophages, as key contributors to the pathogenesis of sepsis-induced cardiac injury (Liu et al., 2025; Yang et al., 2025). Sepsis is characterized by profound dysregulation of inflammatory and immune responses, and macrophages, as pivotal components of the innate immune system, can undergo polarization toward either pro-inflammatory M1 phenotype or the anti-inflammatory M2 phenotype in response to specific microenvironment cues (Wang and Wang, 2023). M1-polarized macrophages release a broad array of pro-inflammatory mediators that drive the progression of sepsis (Chen et al., 2021), whereas, promoting M2 polarization has been shown to facilitate repair of sepsis-induced myocardial damage (Zou et al., 2023). Research findings demonstrate that macrophages not only orchestrate systemic inflammation during sepsis but also directly impair cardiac dysfunction through the secretion of pro-inflammatory cytokines and metabolites (Drosatos et al., 2015). In particular, M1 macrophages produce tumor necrosis factor (TNF-α) and IL-1β, which increase cardiomyocyte apoptosis and reduce contractile performance (Habimana et al., 2020).

It is well-established that metabolic acidosis, which occurs in sepsis, can significantly influence immune cell metabolism and function. Like the tumor microenvironment in cancer, the acidic milieu observed in sepsis may influence immune cell polarization and metabolic reprogramming. In cancer, acidosis has been shown to drive macrophage polarization from a pro-inflammatory M1 phenotype toward an immune-suppressive M2 phenotype, thereby facilitating immune evasion and disease progression. This shift is mediated, at least in part, by lactate accumulation, which activates HIF-1α signaling and engages the GPR132 receptor on macrophages (Cappellesso et al., 2024). In sepsis, the acidic conditions may similarly reprogram macrophages, enhancing the inflammatory response and potentially exacerbating the progression of septic cardiomyopathy. Macrophage-derived lactate and other glycolytic byproducts may exert paracrine effects on cardiomyocyte mitochondrial activity and calcium handling, exacerbating myocardial injury (Mouton et al., 2020; Liu Y. et al., 2021).

These findings not only underscore the central role of macrophages in septic cardiomyopathy but also suggest that modulating their function may provide therapeutic benefit. Consequently, targeting macrophage immunometabolism has emerged as a potential therapeutic strategy for treating sepsis and its complications (Kumar, 2018). Macrophages possess unique metabolic features associated with their functional states, which are referred to as metabolic reprogramming (Liu Y. et al., 2021). Glycolysis, the metabolic pathway responsible for converting glucose into pyruvate, accumulating evidence suggests that glycolytic activity is crucial for macrophage polarization, particularly for M1 macrophages that predominantly depend on glycolysis (Mouton et al., 2020). Hence, several studies have endeavored to inhibit macrophage M1 polarization via blocking glycolysis (Zhai et al., 2021).

This metabolic dependency forms the basis of the emerging concept of immunometabolism, where cellular metabolism directly governs immune cell phenotype and function. Differentiation antagonizing non-protein coding RNA (DANCR), commonly considered an oncogenic lncRNA, is intricately involved in cancer progression (Ghafouri-Fard et al., 2022). DANCR has also been implicated in various inflammatory diseases beyond cancer. For example, it exacerbates intestinal epithelial injury and barrier dysfunction in sepsis via the miR-1306-5p/PLK1 axis (Wang et al., 2021). Furthermore, DANCR expressed in macrophage cells and played a part in the development of coronary artery disease, it promotes lipid accumulation in macrophages, which is closely linked to pro-inflammatory activation (Zhao et al., 2024). DANCR has also been found to regulate glycolysis (Wu et al., 2023), which has previously been reported to be instrumental in regulating macrophage plasticity (Liu Y. et al., 2021; El Kasmi and Stenmark, 2015). Sun et al. demonstrated that modulating macrophage metabolic reprogramming can ameliorate sepsis-induced cardiomyopathy (Sun et al., 2024). Consequently, we propose that DANCR may influence the development of sepsis cardiomyopathy by regulating macrophage glycolytic reprogramming pathways and subsequently influencing macrophage polarization.

## 2 Materials and methods

### 2.1 Cell culture

The THP-1 cell line (CL-0233) and human cardiomyocyte AC16 (CL-0790) were obtained from Procell Corporation (China). The THP-1 cells were cultured in RPMI-1640 medium (PM150110, Procell, China) supplemented with 0.05 mM β-mercaptoethanol (M3148, Merck, Germany), 10% fetal bovine serum (FBS, 164210, Procell, China), and 1% penicillin-streptomycin (PB180120, Procell, China). The AC16 cells were maintained in DMEM/F12 medium (PM150310, Procell, China) supplemented with 10% FBS and 1% penicillin-streptomycin. All cells were grown in an incubator with 5% CO_2_ at 37 °C.

### 2.2 Cell transfection

The entire Insulin-like growth factor 2 mRNA-binding protein 2 (IGF2BP2) or hexokinase 2 (HK2) sequence was inserted into the pcDNA3.1(+) vector (ZK347, Zomanbio, China) to construct the IGF2BP2 overexpression plasmid (oe-IGF2BP2) and HK2 overexpression plasmid (oe-HK2), with the empty vector serving as a negative control (NC). The small interfering RNA (siRNA) sequence targeting DANCR (siDANCR) and scramble (siNC) were designed and synthesized by Ribobio Company (Guangzhou, China). Cells were seeded in 6-well plates at 60%–70% confluency 1 day before transfection. The plasmid (1 μg) or siRNA (50 nM) was delivered to cells using Lipo8000 transfection reagent (C0533, Beyotime, China). The transfection effectiveness was assessed 48 h later using quantitative real-time polymerase chain reaction (qRT-PCR).

### 2.3 Cell treatment

The experiment was conducted in three parts.

First part: THP-1 cells were partitioned into four groups: Control group (THP-1 cells were stimulated with 100 ng/mL phorbol myristate acetate (PMA, HY-18739, MedChemExpress, China) for 24 h (Zhou et al., 2020)), lipopolysaccharides (LPS) group (THP-1 cells post-PMA stimulation were incubated with 50 ng/mL LPS (HY-D1056, MedChemExpress, China) for 48 h (Wang et al., 2023)), LPS + siNC group, and LPS + siDANCR group (THP-1 cells post-PMA and LPS stimulation were transfected with siNC or siDANCR respectively).

Second part: THP-1 cells were partitioned into three groups: LPS + siNC + NC group, LPS + siDANCR + NC group and LPS + siDANCR + HK2. THP-1 cells post-PMA (100 ng/mL, 24 h) and LPS (50 ng/mL, 48 h) stimulation were co-transfected with siNC/siDANCR and oe-HK2/NC.

Third part: THP-1 cells were partitioned into three groups: LPS + NC group, LPS + HK2 group and LPS + HK2 + 2-DG. THP-1 cells post-PMA (100 ng/mL, 24 h) and LPS (50 ng/mL, 48 h) stimulation were transfected with oe-HK2/NC in the presence or absence of glycolysis inhibitor 2-deoxy-d-glucose (2-DG, 10 μL of 10 mM (Qin et al., 2017)). Conditioned medium (CM) was collected from each group of cells and used to culture AC16 cells for 24 h.

### 2.4 qRT-PCR

Nucleus RNA and cytoplasmic RNA were obtained by isolation from cells according to the instructions of the PARIS™ kit (AM 1921, ThermoFisher, United states). The reverse transcription kit (AG11705, AGBIO, China) was utilized to transcribe the RNA procured from an RNA isolation kit (RE-03111, ForeGene, China) into complementary DNA. SYBR Green reagent kit (AG11741, AGBIO, China) was used for fluorescent analysis. We have resorted to the 2^−ΔΔCT^ method to equate the target gene transcript levels to those of the β-actin or U6 (Livak and Schmittgen, 2001). The specific primer sequences for qRT-PCR are delineated in Table 1.

**TABLE 1:** Primers used in this study.

Genes	5′-->3′
DANCR (human) forward	CTTGAGGACCTGACCACACC
DANCR (human) reverse	GTGATCCCAGACAGGTCAGC
GLUT1 (human) forward	ATACTCATGACCATCGCGCTAG
GLUT1 (human) reverse	AAAGAAGGCCACAAAGCCAAAG
HK2 (human) forward	GCCCACCTACGTGTGTGCTA
HK2 (human) reverse	CACCCCACTTCCCATTCCGA
LDHA (human) forward	TAGGCTACAACAGGATTCTAGGTGGAG
LDHA (human) reverse	GTCAGAGGTGGCAGAACTATTTC
TNF-α (human) forward	GCCAGCCTTCATCCACTCTC
TNF-α (human) reverse	GGGAACTGTTGGGGAGAAGG
IL-6 (human) forward	GTCCAGTTGCCTTCTCCCTGG
IL-6 (human) reverse	CCCATGCTACATTTGCCGAAG
IL-1β (human) forward	AACCTCTTCGAGGCACAAGG
IL-1β (human) reverse	AGATTCGTAGCTGGATGCCG
β-actin (human) forward	AGAGCTACGAGCTGCCTGAC
β-actin (human) reverse	AGCACTGTGTTGGCGTACAG
U6 (human) forward	CTCGCTTCGGCAGCACA
U6 (human) reverse	AACGCTTCACGAATTTGCGT

### 2.5 Flow cytometry

For the analysis of macrophage M1 polarization, cell suspensions are collected in EP tubes and then incubated with PE-labelled CD86 (374205; Biolgend, United states), as well as APC-labelled CD206 (321109; Biolgend, United states) for a period of 30 min at 4 °C in darkness. Following washing, cells are resuspended in phosphate-buffered saline (PBS, E607008, Sangon, China) containing 3% FBS and analyzed via flow cytometry (CytoFlex, Beckman, United states). A stepwise gating strategy was applied: (1) forward scatter (FSC) vs. side scatter (SSC) to select the cell population of interest, (2) FSC-A vs. FSC-H to exclude doublets, and (3) Fluorescence intensities of CD86 and CD206 were quantified using mean fluorescence intensity (MFI) values, with gating boundaries defined based on corresponding isotype controls. For the detection of apoptosis, cells are washed with PBS and sequentially exposed to Annexin V-FITC and PI Staining Solution (40302ES20; Yeasen, China). Following a 10-min incubation time in darkness, binding buffer is introduced to the cell suspension before flow cytometry analysis. A stepwise gating strategy was applied: (1) forward scatter (FSC) vs. side scatter (SSC) to identify the main cell population and exclude debris, (2) FSC-A vs. FSC-H to remove doublets, and (3) Annexin V vs. PI plots to distinguish apoptotic and necrotic populations, with quadrants defined using unstained and single-stained controls. Early apoptotic cells were defined as Annexin V^+^/PI^−^, late apoptotic cells as Annexin V^+^/PI^+^. Total apoptosis rate (%) was calculated as the sum of early and late apoptotic populations. Representative gating plots are provided in Supplementary Figures S1–S3.

### 2.6 Enzyme-linked immunosorbent assay (ELISA)

The levels of pro-inflammatory mediator TNF-α, IL-1β, and IL-6 in cell supernatant were measured using ELISA kits (E-EL-H0109/E-EL-H0149/E-EL-H6156, Elabscience, China). THP-1 cells were plated at 1 × 10^6^ cells/well in 6-well plates. After the above treatment, collect the cells, centrifuge at 1000 revolutions per minute for 20 min, and separate the supernatant. Standard solution and sample diluents were meticulously prepared as per the manufacturer’s guidelines. Following the addition of samples and standards into the respective wells, they were incubated at 37 °C for 90 min. Subsequently, biotinylated antibodies, horseradish peroxidase (HRP) enzyme conjugates, and tetramethylbenzidine (TMB) substrate solutions were sequentially introduced into the wells, with each subsequent step necessitating a thorough washing of the test well prior to reagent addition, followed by an allotted incubation period post-reagent introduction. Lastly, the well was supplemented with the appropriate stop solution, and the absorbance reading at 450 nm was obtained via a microplate reader (DeTiebio, China). The standard curve for inflammatory cytokine detection is shown in Supplementary Figure S4.

### 2.7 Detection of glucose lactate and ATP

Utilizing the Glucose Uptake Assay Kit (23500, xabiolite, China), Lactate Assay Kit (KTB1100, Abbkine, China) and ATP Content Assay Kit (KTB1016, Abbkine, China), the determination of glucose uptake, lactate production levels, as well as ATP content was conducted in accordance with the specifications stipulated by their manufacturers.

### 2.8 Real-time cell metabolism assay

Seahorse XFp analyzer was used to detect the extracellular acidification rate (ECAR) and the oxygen consumption rate (OCR) as previously described (Plitzko and Loesgen, 2018). THP-1-derived macrophages were seeded at 5 × 10^4^ cells/well in Seahorse XF24 microplates and incubated overnight. Before the assay, cells were washed and incubated in Seahorse XF assay medium (supplemented with 10 mM glucose, 1 mM pyruvate, and 2 mM glutamine) for 1 h at 37 °C in a CO_2_-free incubator. For OCR measurement, the Mito Stress Test protocol was followed with sequential injections of oligomycin (10 μM), FCCP (5 μM), and rotenone/antimycin A (5 μM each). For ECAR measurement, the Glycolysis Stress Test was applied, including glucose (10 mM), oligomycin (10 μM), and 2-deoxy-D-glucose (50 mM) injections. Each condition was tested with 3 technical replicates per group and at least 3 biological replicates. ECAR reflects glycolytic activity, while OCR reflects mitochondrial respiration. Basal respiration and maximal respiration were calculated using Wave 2.6.1 software (Agilent) according to the manufacturer’s protocol. Glycolysis rate and glycolytic capacity were derived from ECAR changes following sequential compound injections. All Seahorse parameters were normalized to total cellular protein content (μg per well) to correct for variations in cell number and viability. These calculation procedures and normalization criteria have been added to ensure methodological transparency and reproducibility. Changes in ECAR and OCR were interpreted in the context of macrophage polarization and glycolytic reprogramming, which directly impact inflammatory response and paracrine effects on cardiomyocytes.

### 2.9 Cell viability

Cardiomyocyte AC16 cells were seeded in 6-well plates at a density of 1 × 10^5^ cells/well and allowed to adhere overnight. After transfection and treatment, THP-1-derived macrophages were harvested, and CM was collected after 24 h of incubation. The CM was centrifuged at 1000 rpm for 10 min to remove cells and debris and subsequently added to cardiomyocyte cultures (1:1 ratio with fresh medium) for 24 h. No direct co-culture or physical contact between the cell types was implemented; rather, a conditioned medium-based model was used to evaluate paracrine effects. To discern the impact of macrophage polarization on cardiomyocyte, we quantified the viability of cardiomyocyte co-cultured with macrophages. Cardiomyocytes were incubated with a volume of 10 μL Cell Counting Kit-8 (CCK-8, D002-1, Research-Bio, China), for a period of two hours. Subsequently, the optical density at a wavelength of 450 nm was assessed using a microplate reader. Viability was calculated as: % Viability = (OD treated/OD control) × 100%, where OD control is the absorbance of untreated control wells.

### 2.10 Bioinformatics analysis

LncATLAS database (https://lncatlas.crg.eu/?tdsourcetag=s_pcqq_aiomsg) was used to predict DANCR location in cells. StarBase (https://rnasysu.com/encori/index.php) predicts that DANCR may promote HK2 expression through IGF2BP2.

### 2.11 RNA immunoprecipitation (RIP)

RIP kit (P1801S, Beyotime, China) was employed to validate the relationship between DANCR and IGF2BP2. To summarize, following a series of washings, Protein A/G Agarose is pre-incubated with either anti-IgG antibodies or anti-IGF2BP2 antibodies (11601-1-AP, Proteintech, China) for 1 h at 4 °C, followed by centrifugation at 1000 *g* for 1 min to eliminate the supernatant at 4 °C. The cells are lysed using lysis solution and subsequently added to the antibody-bound Protein A/G Agarose, which is then incubated at 4 °C for 4 hours. Subsequently, the mixture is subjected to washes utilizing wash buffer and elution buffer. Post purification of RNA, HK-2 expression is determined through qRT-PCR analysis.

### 2.12 RNA fluorescence *in situ* hybridization (RNA-FISH) and immunofluorescence co-staining

THP-1 cells were seeded on poly-L-lysine-coated coverslips, differentiated with PMA, and stimulated with LPS. Cells were fixed with 4% paraformaldehyde for 15 min, permeabilized with 0.5% Triton X-100 for 10 min and incubated with Cy3-labeled antisense probes targeting DANCR (RiboBio, China) overnight at 37 °C in a humid chamber. After stringent washes, cells were blocked with 5% BSA and incubated with anti-IGF2BP2 antibody for 2 h at room temperature, followed by Alexa Fluor 488-conjugated secondary antibody (A-11008, Invitrogen, United states) for 1 h. Nuclei were counterstained with DAPI, and coverslips were mounted using antifade mounting medium. Fluorescence signals were visualized using a confocal microscope.

### 2.13 RNA stability assay

Based on the preceding delineation, RNA stability analysis has been executed (Lang et al., 2021). In essence, cells were cultivated overnight in a 6 well dish. Subsequently, 5 μg/mL actinomycin D (M4881, Abmole, China) was added to the cells to inhibit gene transcription for varied durations. Subsequently, RNA was extracted and quantified by qRT-PCR.

### 2.14 Western blotting

Cells were lysed in RIPA buffer (P0013B, Beyotime, China) supplemented with protease and phosphatase inhibitors. Protein concentrations were determined using the BCA assay (P0010, Beyotime, China). Equal amounts of protein were separated by 10% SDS-PAGE and transferred to PVDF membranes. Membranes were blocked with 5% non-fat milk for 1 h at room temperature and incubated overnight at 4 °C with the following primary antibodies: anti-HK2 (1:1000, #2867, Cell Signaling Technology, United states) and anti-β-actin (1:5000, ab6276, Abcam, United Kingdom). After washing, membranes were incubated with HRP-conjugated secondary antibodies (1:5000, ZB-2301, ZSGB-BIO, China) for 1 h. Signals were detected using ECL reagent (P0018FS, Beyotime, China) and imaged with a ChemiDoc system (Bio-Rad, United states). Band intensity was quantified using ImageJ and normalized to β-actin.

### 2.15 Statistical analysis

All experiments were performed with at least three independent biological replicates, defined as separately cultured and treated samples from distinct passages or thawing of cells. Technical replicates (typically 3 wells per condition) were used for each biological replicate when measuring assays such as qRT-PCR, ELISA, and metabolic flux. Statistical analysis was conducted using GraphPad Prism 8.0. The measurement data were presented as mean ± standard deviation. One-way or two-way ANOVA was used for comparative analysis among three or more groups. For two-group comparisons, Student’s t-test was applied. Differences with *P* < 0.05 were deemed statistically significant.

## 3 Results

### 3.1 DANCR silencing suppresses M1 polarization and glycolysis in macrophages and protects cardiomyocytes from damage

**FIGURE 1:**
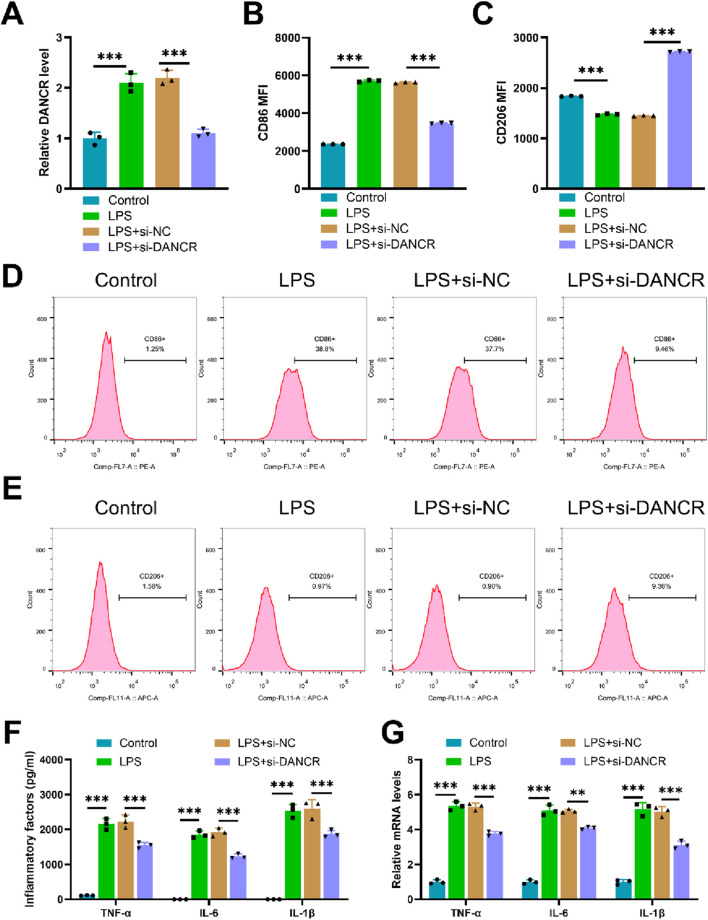
Effect of siDANCR on M1 polarization of macrophages. The THP-1 cells were partitioned into four groups: Control group (100 ng/mL phorbol myristate acetate (PMA) to stimulate THP-1 cells for 24 h), lipopolysaccharide (LPS) group (THP-1 cells post-PMA stimulation were incubated with 50 ng/mL LPS for 48 h), LPS + siNC group, and LPS + siDANCR group (THP-1 cells post-PMA and LPS stimulation were transfected with siNC or siDANCR respectively). **(A)** Quantitative real-time PCR (qRT-PCR) was used to detect the transfection efficiency of siDANCR. **(B-D)** Representative flow cytometry images and MFI of CD86^+^ macrophages. **(C,E)** Representative flow cytometry images and MFI of CD206+ macrophages. **(F)** Inflammatory factor (TNF-α, IL-6, and IL-1β) levels in cell culture media were detected by enzyme-linked immunosorbent assay (ELISA). **(G)** qRT-PCR was used to detect the expression of inflammatory factors (TNF-α, IL-6, and IL-1β) in various groups of cells. Data are presented as mean ± SD of three independent biological replicates (n = 3). Statistical analysis: one-way ANOVA with Tukey’s *post hoc* test; *P < 0.05, **P < 0.01, ***P < 0.001.

**FIGURE 2:**
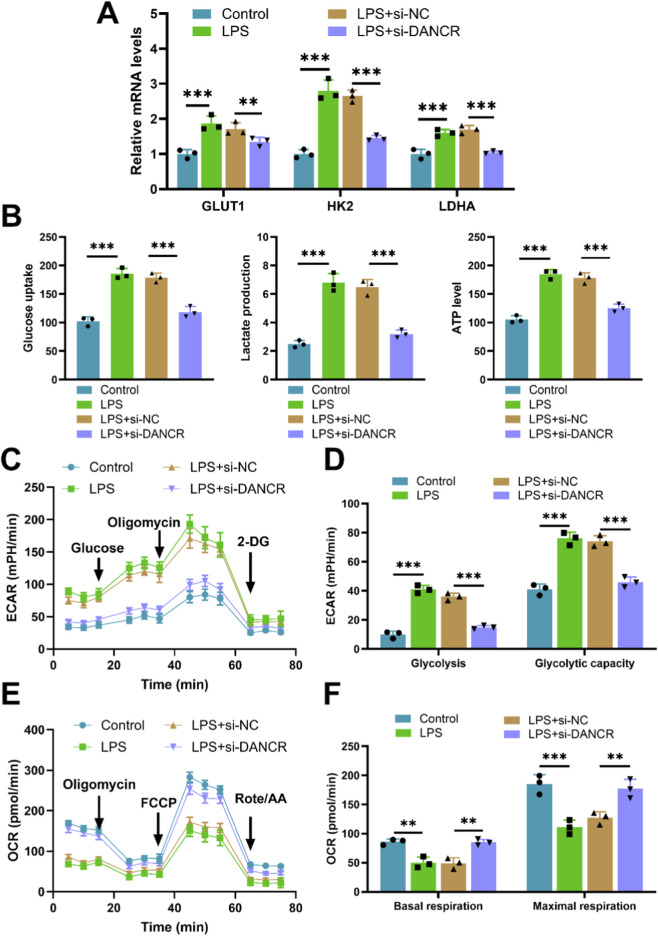
Effect of siDANCR on glycolysis of macrophages. The THP-1 cells were partitioned into four groups: Control group (100 ng/mL phorbol myristate acetate (PMA) to stimulate THP-1 cells for 24 h), lipopolysaccharide (LPS) group (THP-1 cells post-PMA stimulation were incubated with 50 ng/mL LPS for 48 h), LPS + siNC group, and LPS + siDANCR group (THP-1 cells post-PMA and LPS stimulation were transfected with siNC or siDANCR respectively). **(A)** Expression of glycolytic enzymes (GLUT1, HK2 and LDHA) in cells was detected by quantitative real-time PCR (qRT-PCR). **(B)** Detection of glucose intake, lactate production, and ATP content using kits. **(C–F)** Seahorse XFp analyzer was used to detect the extracellular acidification rate (ECAR) and oxygen consumption rate (OCR). Data are presented as mean ± SD of three independent biological replicates (n = 3). Statistical analysis: one-way ANOVA with Tukey’s *post hoc* test; *P < 0.05, **P < 0.01, ***P < 0.001.

Upon exposure to LPS, M0 macrophages demonstrated increased expression of the gene DANCR. Conversely, siDANCR transfection successfully mitigated this trend, indicating successful silencing of DANCR (Figure 1A, *P* < 0.001). Subsequently, we evaluated the influence of DANCR silencing on macrophage polarization towards an M1 phenotype. Predictably, LPS stimulation increased the expression of the M1 macrophage marker CD86 and decreased the expression of the M2 marker CD206. (Figures 1B–E, P < 0.001). and proinflammatory cytokines (TNF-α, IL-6, and IL-1β) (Figures 1F–G, P < 0.001). However, siDANCR reversed the stimulatory effect of LPS on macrophage M1 polarization (Figures 1B–E, P < 0.01). To assess the role of DANCR in macrophage glycolysis, we examined the expression of key glycolytic enzymes (GLUT1, HK2 and LDHA). LPS was observed to stimulate mRNA levels of these glycolytic enzymes (Figure 2A, *P* < 0.001), in addition to augmenting glucose uptake, lactate production, and ATP content (Figure 2B, *P* < 0.001). A real-time cell metabolism assay confirmed that LPS promoted ECAR while suppressing OCR (Figures 2C–F). Nevertheless, all these effects of LPS were counteracted by siDANCR (Figures 2A–F, P < 0.01). These findings suggest that DANCR silencing suppresses macrophage M1 polarization and glycolysis.

**FIGURE 3:**
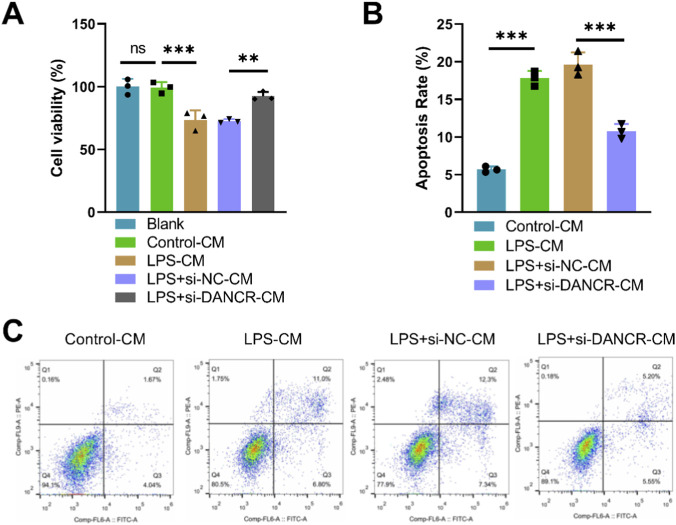
Effects of conditioned medium of DANCR-deficient macrophages on viability and apoptosis of cardiomyocytes. The cardiomyocytes were treated with conditioned medium (CM) from THP-1 cells partitioned into four groups: Control-CM group (CM from 100 ng/mL phorbol myristate acetate (PMA) to stimulate THP-1 cells for 24 h), lipopolysaccharide (LPS) group (CM from THP-1 cells post-PMA stimulation were incubated with 50 ng/mL LPS for 48 h), LPS + siNC group, and LPS + siDANCR group (CM from THP-1 cells post-PMA and LPS stimulation were transfected with siNC or siDANCR respectively). **(A)** CCK-8 was used to detect the cardiomyocytes viability. **(B,C)** Flow cytometry was used to detect the cardiomyocytes apoptosis. Data are presented as mean ± SD of three independent biological replicates (n = 3). Statistical analysis: one-way ANOVA with Tukey’s *post hoc* test; *P < 0.05, **P < 0.01, ***P < 0.001.

Upon treating cardiomyocytes with conditioned media from various macrophage subsets, we observed a substantial decrease in cardiomyocyte viability (Figure 3A, *P* < 0.05) and an increase in apoptosis (Figures 3B,C,P < 0.05) when these cells were exposed to conditioned media from LPS-stimulated macrophages. Interestingly, compared to the LPS + siNC-CM group, cardiomyocyte viability significantly increased while apoptosis diminished in the LPS + siDANCR-CM group (Figures 3A–C, P < 0.05). This revelation signifies that macrophages exhibiting low DANCR expression can provide cardio-protection against damage.

### 3.2 DANCR facilitates HK2 expressions by regulating the binding of IGF2BP2 and HK2

**FIGURE 4:**
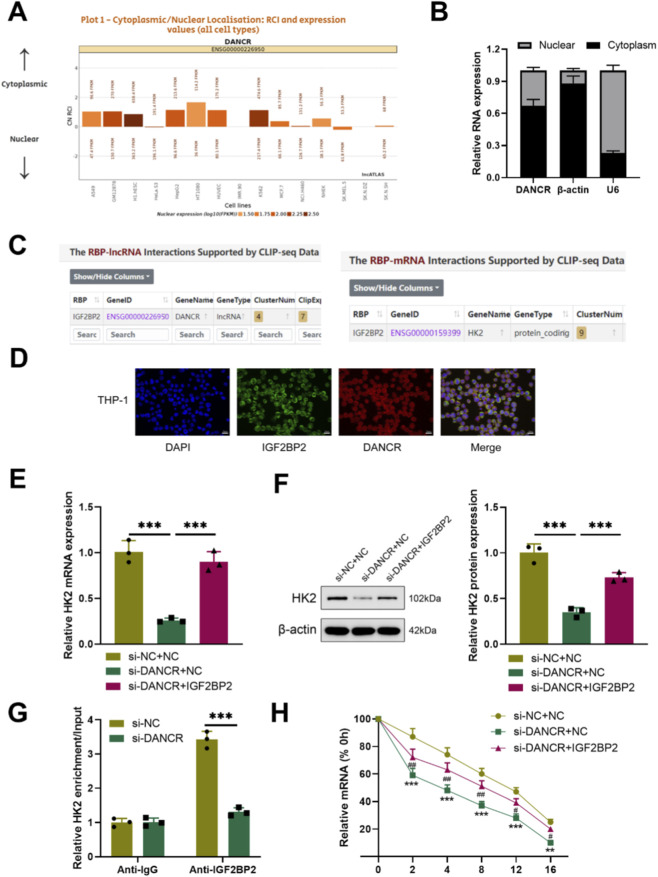
DANCR regulates IGF2BP2 and HK-2 interactions in the cytoplasm. **(A)** LncATLAS database (https://lncatlas.crg.eu/?tdsourcetag=s_pcqq_aiomsg) was used to predict DANCR location in cells. **(B)** Quantitative real-time PCR (qRT-PCR) detection of DANCR levels in cytoplasm and nucleus. **(C)** StarBase (https://rnasysu.com/encori/index.php) predicts that DANCR may promote HK2 expression through IGF2BP2. **(D)** RNA-FISH and immunofluorescence co-localization of DANCR and IGF2BP2 in LPS-stimulated THP-1 macrophages. **(E)** Cells were co-transfected with siNC/siDANCR and oe-IGF2BP2/NC and then the HK-2 expression in cells was determined by qRT-PCR. **(F)** Cells were co-transfected with siNC/siDANCR and oe-IGF2BP2/NC and then the HK-2 expression in cells was determined by Western blot. **(G)** RNA immunoprecipitation (RIP) assay was used to verify the relationship between DANCR and HK2. **(H)** Transfected cells were treated with actinomycin D for the indicated times, and then mRNA expression levels were examined by qRT-PCR. Data are presented as mean ± SD of three independent biological replicates (n = 3). Statistical analysis: one-way ANOVA with Tukey’s *post hoc* test and Student’s t-test; *P < 0.05, **P < 0.01, ***P < 0.001.

To elucidate the mechanism by which DANCR modulates glucose metabolism, we initially identified its cellular localization. LncATLAS database prediction indicated that DANCR primarily resides in the cytoplasm (Figure 4A). Consistent with this finding, Cytoplasm/nuclear RNA assays confirmed a predominant cytoplasmic distribution of DANCR within THP-1 cells (Figure 4B). Concurrently, starBase predicted DANCR may regulate HK2 via IGF2BP2 (Figure 4C). RNA-FISH combined with immunofluorescence confirmed that DANCR is predominantly localized in the cytoplasm and exhibits significant co-localization with IGF2BP2 in LPS-stimulated THP-1 macrophages (Figure 4D). To establish this correlation, we evaluated HK2 mRNA and protein levels and observed reduced expression of HK2 in siDANCR-transfected cells (Figures 4E,F, P < 0.001). Interestingly, over-expression of IGF2BP2 effectively reversed the inhibitory effect of siDANCR on HK2 (Figure 4E,F, *P* < 0.001). RIP experiments demonstrated enrichment of HK2 onto IGF2BP2 protein, which was reversed by siDANCR (Figure 4G, *P* < 0.001), suggesting that siDANCR can interfere with the binding of IGF2BP2 and HK2. Furthermore, siDANCR induced destabilization of HK2 mRNA, which was counteracted by oe-IGF2BP2 (Figure 4H, *P* < 0.05). These findings indicate that DANCR facilitates HK2 expressions by regulating the binding of IGF2BP2 and HK2.

### 3.3 HK2 overexpression reversed the effects of siDANCR on M1 polarization and glycolysis in macrophages and cardiomyocytes damage

**FIGURE 5:**
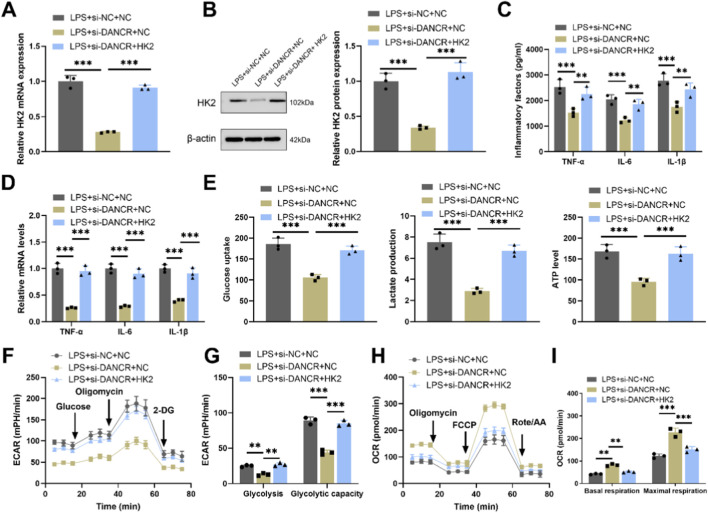
HK2 overexpression reverses the effects of DANCR deficiency on macrophage polarization and glycolysis. The THP-1 cells were partitioned into three groups: lipopolysaccharide (LPS) + siRNA negative control (siNC) + NC group, LPS + siDANCR + NC group and LPS + siDANCR + HK2. THP-1 cells post-phorbol myristate acetate (PMA) (100 ng/mL, 24 h) and LPS (50 ng/mL, 48 h) stimulation were co-transfected with siNC/siDANCR and oe-HK2/NC. **(A,B)** Quantitative real-time PCR (qRT-PCR) and Western blot (WB) were used to detect the expressions of HK2. **(C)** Inflammatory factor (TNF-α, IL-6, and IL-1β) levels in cell culture media were detected by ELISA. **(D)** qRT-PCR was used to detect the expression of inflammatory factors (TNF-α, IL-6, and IL-1β) in various groups of cells. **(E)** Detection of glucose intake, lactate production, and ATP content using kits. **(F–I)** Seahorse XFp analyzer was used to detect the extracellular acidification rate (ECAR) and oxygen consumption rate (OCR). Data are presented as mean ± SD of three independent biological replicates (n = 3). Statistical analysis: one-way ANOVA with Tukey’s *post hoc* test; *P < 0.05, **P < 0.01, ***P < 0.001.

**FIGURE 6:**
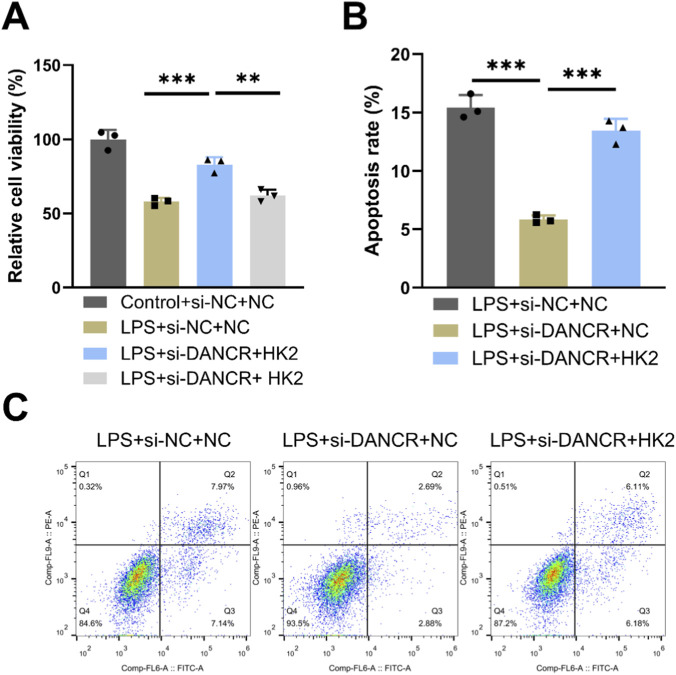
Effects of HK2 overexpression on DANCR-deficient macrophages. The THP-1 cells were partitioned into three groups: lipopolysaccharide (LPS) + siRNA negative control (siNC) + NC group, LPS + siDANCR + NC group and LPS + siDANCR + HK2. THP-1 cells post-phorbol myristate acetate (PMA) (100 ng/mL, 24 h) and LPS (50 ng/mL, 48 h) stimulation were co-transfected with siNC/siDANCR and oe-HK2/NC. Conditioned medium for each of the above groups of cells was used to culture cardiomyocytes. **(A)** CCK-8 was used to detect the cell viability of cardiomyocytes. **(B,C)** Flow cytometry detects apoptosis. Data are presented as mean ± SD of three independent biological replicates (n = 3). Statistical analysis: one-way ANOVA with Tukey’s *post hoc* test; *P < 0.05, **P < 0.01, ***P < 0.001.

To investigating whether the effects of DANCR on macrophage polarization and glycolysis are mediated through HK2, we conducted a series of rescue experiments. Firstly, co-transfection of siDANCR and HK2 in cells successfully reversed the inhibitory effect of siDANCR on HK2 expression (Figures 5A,B, P < 0.001). Subsequently, we observed that the introduction of oe-HK2 not only reverted the regulation of siDANCR on inflammatory factor secretion (Figures 5C,D, P < 0.001), glucose uptake, lactate production, ATP content (Figure 5E, *P* < 0.001), ECAR, and OCR (Figures 5F–I) but also counteracted the stimulatory effect of siDANCR on cardiomyocyte viability and the inhibitory effect on cell apoptosis (Figures 6A–C, P <0.05). This suggests a potential role for DANCR in influencing macrophage polarization and glycolysis as well as cardiomyocyte vitality through its regulation of HK2.

### 3.4 2-DG reversed the effects of oe-HK2 on M1 polarization and glycolysis in macrophages and cardiomyocytes damage

**FIGURE 7:**
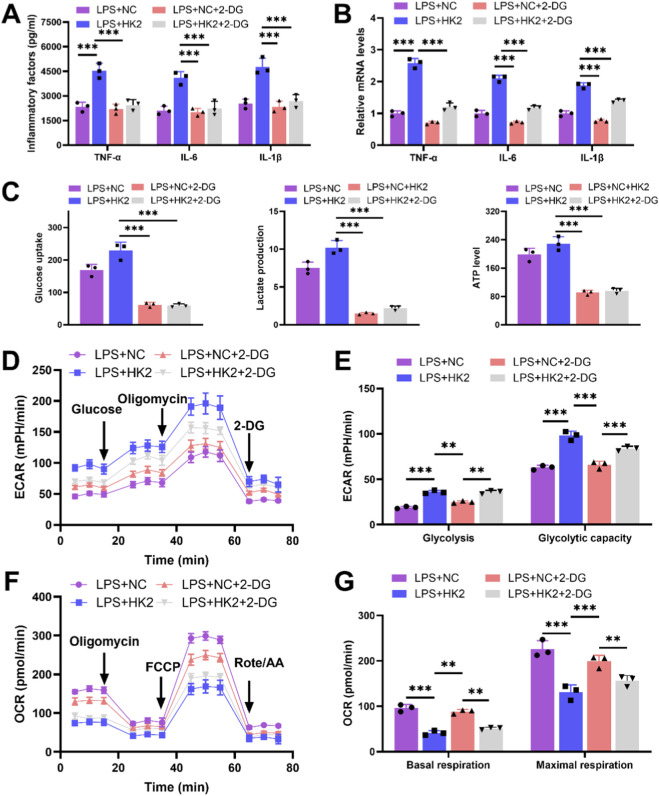
2-deoxy-D-glucose (2-DG) reverses the effects of HK-2 overexpression on macrophage polarization and glycolysis. The THP-1 cells were partitioned into three groups: lipopolysaccharide (LPS) + negative control (NC) group, LPS + HK2 group, LPS + NC + 2-DG and LPS + HK2 + 2-DG. THP-1 cells post-phorbol myristate acetate (PMA) (100 ng/mL, 24 h) and LPS (50 ng/mL, 48 h) stimulation were transfected with oe-HK2/NC in the presence or absence of 2-DG (glycolysis inhibitor). **(A)** Inflammatory factor (TNF-α, IL-6, and IL-1β) levels in cell culture media were detected by enzyme-linked immunosorbent assay (ELISA). **(B)** Quantitative real-time PCR (qRT-PCR) was used to detect the expression of inflammatory factors (TNF-α, IL-6, and IL-1β) in various groups of cells. **(C)** Detection of glucose intake, lactate production, and ATP content using kits. **(D–G)** Seahorse XFp analyzer was used to detect the extracellular acidification rate (ECAR) and oxygen consumption rate (OCR). Data are presented as mean ± SD of three independent biological replicates (n = 3). Statistical analysis: one-way ANOVA with Tukey’s *post hoc* test; *P < 0.05, **P < 0.01, ***P < 0.001.

**FIGURE 8:**
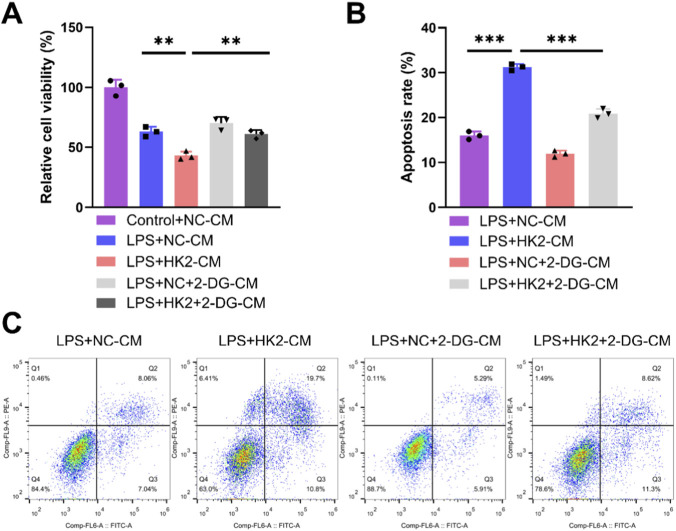
2-deoxy-D-glucose (2-DG) reverses the effects of HK-2 overexpressed macrophages on cardiomyocyte. The cardiomyocytes were treated with conditioned medium (CM) from THP-1 cells partitioned into three groups: lipopolysaccharide (LPS) + negative control (NC)-CM group, LPS + HK2-CM group and LPS + HK2 + 2-DG-CM. THP-1 cells post-PMA (100 ng/mL, 24 h) and LPS (50 ng/mL, 48 h) stimulation were transfected with oe-HK2/NC in the presence or absence of 2-DG (glycolysis inhibitor). Conditioned medium for each of the above groups of cells was used to culture cardiomyocytes. **(A)** CCK-8 was used to detect the cell viability of cardiomyocytes. **(B,C)** Flow cytometry detects apoptosis. *P < 0.05, **P < 0.01 vs. LPS + NC-CM or LPS + HK2-CM. Data are presented as mean ± SD of three independent biological replicates (n = 3). Statistical analysis: one-way ANOVA with Tukey’s *post hoc* test; *P < 0.05, **P < 0.01, ***P < 0.001.

To verify whether the effect of DANCR/HK2 on macrophage polarization and cardiomyocyte injury is related to glycolysis, we employed a glycolytic inhibitor 2-DG, to obstruct glycolysis. Initially, the application of 2-DG mitigated oe-HK2’s stimulatory influence on pro-inflammatory cytokine secretion (Figures 7A,B, P <0.001), glucose uptake, lactate production, and ATP generation (Figure 7C, *P* < 0.001), concurrently reversing oe-HK2’s regulation over ECAR and OCR (Figures 7D–G). Furthermore, 2-DG can restore the adverse effects of HK2 overexpressing macrophage-conditioned media on cardiomyocyte viability, while also inhibiting its pro-apoptotic effect (Figures 8A–C, P <0.05). This suggests that DANCR/HK2 may regulate macrophage glycolysis to influence macrophage polarization and cardiomyocyte injury.

## 4 Discussion

In this study, we systematically explored the role of the DANCR in the pathogenesis of septic cardiomyopathy and revealed its contribution to macrophage M1 polarization via glycolytic reprogramming. THP-1 cells, a widely used human monocytic leukemia cell line, were chosen to model macrophage polarization and to explore the associated immune responses. While THP-1 cells are derived from a leukemia patient, they are commonly used to study macrophage function, including polarization and inflammatory responses. *In vitro* experiments demonstrated that silencing DANCR attenuated inflammatory cytokine production and suppressed glycolysis in LPS-stimulated macrophages, thereby improved cardiomyocyte viability. Mechanistically, we identified that DANCR promotes HK2 expression by enhancing IGF2BP2-mediated mRNA stabilization. Rescue experiments using HK2 overexpression and glycolysis inhibition further confirmed the critical role of the DANCR/IGF2BP2/HK2 axis in macrophage-driven myocardial injury. These findings highlight a novel immunometabolism mechanism underlying septic cardiomyopathy and suggest DANCR as a potential therapeutic target.

Non-coding RNAs (ncRNAs), including miRNAs and lncRNAs, play key roles in immune regulation and metabolic reprogramming. miRNAs can reshape macrophage metabolism by targeting glycolytic enzymes and transporters (Virga et al., 2021a). In sepsis, hypoxia-induced miR-210 and miR-21 promote glycolysis and inflammation, exacerbating tissue injury (Virga et al., 2021b; De Melo et al., 2021). Previously, DANCR has been reported to have substantial contributions to cancer progression (Yuan et al., 2024), yet study has demonstrated its protective role in hypoxia-induced myocardial infarction (Qiu et al., 2020). However, currently, as an lncRNA, due to the diverse downstream regulatory mechanisms of DANCR, its function in disease exhibits significant heterogeneity (Zhang Z. et al., 2021). Several studies have highlighted the detrimental role of DANCR in sepsis-related disorders. For instance, Wang et al. found that DANCR aggravates intestinal epithelial injury and barrier dysfunction in sepsis via the miR-1306-5p/PLK1 axis, thereby contributing to systemic inflammation and organ failure (Wang et al., 2021). Moreover, Zhao et al. reported that DANCR promotes lipid accumulation in macrophages, which may exacerbate metabolic dysregulation and inflammation during sepsis (Zhao et al., 2024). These findings suggest that DANCR may act as a pro-inflammatory regulator across multiple sepsis-associated organs, not just the myocardium. The role of DANCR in cardiac pathology has also been observed in non-septic models. Huang et al. demonstrated that DANCR knockdown attenuated Ang II-induced cardiac hypertrophy and fibrosis in H9C2 cardiomyoblasts, thereby mitigating heart failure progression (Huang and Huang, 2023). Additionally, Qiu et al. showed that DANCR alleviated hypoxia-induced damage in H9C2 cells via upregulation of HIF-1α (Qiu et al., 2020), suggesting that the role of DANCR in cardiac injury may be context-dependent and vary based on downstream effectors and disease models. In this study, we initially dissected the role of DANCR in macrophage polarization, glycolysis, and LPS-induced cardiomyocyte damage. It is noteworthy that research established that silencing DANCR could diminish the expression of inflammatory factors in rats with colitis (Zhang X. et al., 2021), while overexpression of DANCR could augment inflammation following spinal cord injury (Shi et al., 2024), indicating a pro-inflammatory role for DANCR in disease. Consistently, we discovered siDANCR blunted the expression of inflammatory factors in LPS-induced macrophages. Currently, there are no studies reporting the regulation of DANCR on macrophage polarization, but Zhao et al. found that DANCR could stimulate lipid accumulation in macrophages (Zhao et al., 2024). Lipid metabolism can influence macrophage polarization, particularly fatty acid production is associated with the production of LPS-induced cytokines in M1 macrophages (Vassiliou and Farias-Pereira, 2023). Therefore, we observed that siDANCR suppressed macrophage M1 polarization. Furthermore, Shi et al. reported that DANCR could upregulate HK2 to activate anaerobic glycolysis in colon cancer (Shi et al., 2020). Wang et al. reported that DANCR enhanced glucose uptake and ECAR in prostate cancer by upregulating LDHA (Wang and Chen, 2022). Consistent with these findings, we observed that siDANCR downregulated the expression of glycolytic enzymes, inhibited glucose uptake, lactate and ATP production. siDANCR also suppressed ECAR, while enhancing the OCR, collectively indicating an inhibitory effect on glycolysis in macrophages. Furthermore, CM from siDANCR-transfected macrophages enhanced AC16 cardiomyocyte viability and reduced apoptosis, suggesting that DANCR silencing may help mitigate the progression of septic cardiomyopathy.

Subsequently, we examined the downstream regulatory mechanism of DANCR and discovered that siDANCR can hinder the association between IGF2BP2 and HK2, subsequently impeding the stability of HK2. HK2 is a rate-limiting enzyme in the process of aerobic glycolysis, and overexpression of it can enhance the efficiency of aerobic glycolysis (Liu et al., 2023). Regulation of HK2 mRNA stability often involves interactions between RNA-binding proteins (RBPs) and their target transcripts. IGF2BP2, also known as IMP2, is an RBP that plays a key role in post-transcriptional gene regulation. Notably, IGF2BP2 has been shown to stabilize HK2 mRNA, thereby promoting the Warburg effect and driving the progression of oral squamous cell carcinoma (Xu et al., 2022). Furthermore, the IGF2BP2 protein also augments the stability of HK2 mRNA by forming a complex with lncRNA CASC9 (Liu H. et al., 2021). This mechanism is also observed here, where DANCR can stimulate the binding of IGF2BP2 to HK2 and thereby stabilize HK2 expression.

Our findings align with previous studies that emphasize the pathological role of M1 macrophages in septic cardiomyopathy. Macrophage-derived cytokines and metabolic products have been implicated in promoting cardiomyocyte apoptosis and metabolic dysregulation. By using conditioned medium from LPS-stimulated macrophages, we simulated this inflammatory microenvironment and demonstrated that DANCR knockdown in macrophages alleviates cardiomyocyte injury, highlighting a key paracrine mechanism.

To probe into whether the effectiveness of DANCR on septic cardiomyopathy is contingent upon HK2 and glycolysis, we separately employed oe-HK2 and glycolytic inhibitors. DANCR has been attested to augment the glycolytic process (Wu et al., 2023; Shi et al., 2020). The augmentation of glycolytic metabolism can stimulate the progression of septic cardiomyopathy, which has been substantiated in sepsis mice, and the utilization of 2-DG can notably ameliorate cardiac dysfunction and survival outcomes in sepsis mice (Zheng et al., 2017). Furthermore, Yuan et al. reported that inhibiting HK2 transcription could curtail glycolysis and proinflammatory cytokine secretion in macrophages, thereby preventing sepsis (Yuan et al., 2022). Consistent with previous studies, we found that HK2 overexpression reversed the effects of DANCR silencing on M1 polarization, glycolysis in macrophages and cardiomyocytes injury, whereas treatment with 2-DG reversed the effects of HK2 overexpression. These findings indicate that DANCR may promote macrophage polarization toward the M1 phenotype and contribute to the development of septic cardiomyopathy through regulating macrophage glycolysis.

While we recognize the value of validating our findings in an *in vivo* model such as the cecal ligation and puncture (CLP)-induced sepsis model, our current study was designed as a mechanistic exploration using a well-established *in vitro* macrophage–cardiomyocyte system. The use of this model allows precise dissection of molecular interactions (DANCR/IGF2BP2/HK2 axis) before proceeding to animal studies. In future work, we aim to extend these findings by establishing a CLP-induced septic cardiomyopathy mouse model to validate the therapeutic potential of targeting DANCR *in vivo*. Furthermore, we intend to explore the possibility of using DANCR-targeting approaches, such as siRNA delivery or small molecule modulators, to evaluate their efficacy in attenuating cardiac injury during sepsis. Elucidating additional components of the immunometabolism regulatory network will also help in identifying synergistic targets for intervention.

## 5 Conclusion

This study highlights the significant role of DANCR in septic cardiomyopathy. DANCR exacerbates LPS-induced myocardial injury by inducing macrophage M1 polarization through stimulating macrophage glycolysis. This pro-glycolytic effect is linked to the stabilization of HK2 mediated by the RNA-binding protein IGF2BP2. Targeting DANCR may therefore represent a promising therapeutic strategy for septic cardiomyopathy. Future studies, particularly *in vivo* animal models, are warranted to further validate these findings.

## Data Availability

The raw data supporting the conclusions of this article will be made available by the authors, without undue reservation.
