# Supplementary material for: DANCR promotes septic cardiomyopathy by enhancing macrophage glycolytic reprogramming via the IGF2BP2/HK2 axis
Source: Front Cell Dev Biol. 2025 Nov 27;13:1628915. doi: 10.3389/fcell.2025.1628915 (PMC12695775; doi:10.3389/fcell.2025.1628915)
Supplement: Supplementary file 1 [file DataSheet1.docx]

**Supplementary Figures**


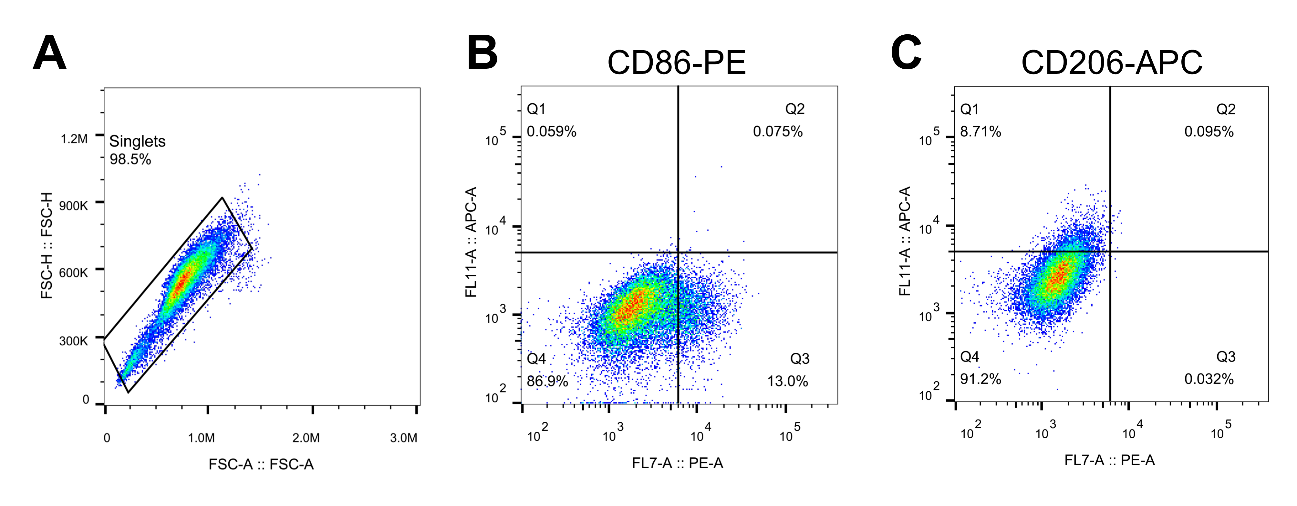
 **Figure S1. Flow cytometric analysis of macrophage surface markers.** (A) Single cells were gated based on FSC-A vs. FSC-H to exclude doublets and debris, with singlets accounting for 98.5% of the events. (B) M1 macrophage marker CD86 was detected using PE-conjugated antibody, with 13.0% of the cells positive for CD86. (C) M2 macrophage marker CD206 was detected using APC-conjugated antibody, with only 0.095% of the cells expressing CD206. These results indicate a predominantly M1-polarized macrophage population.


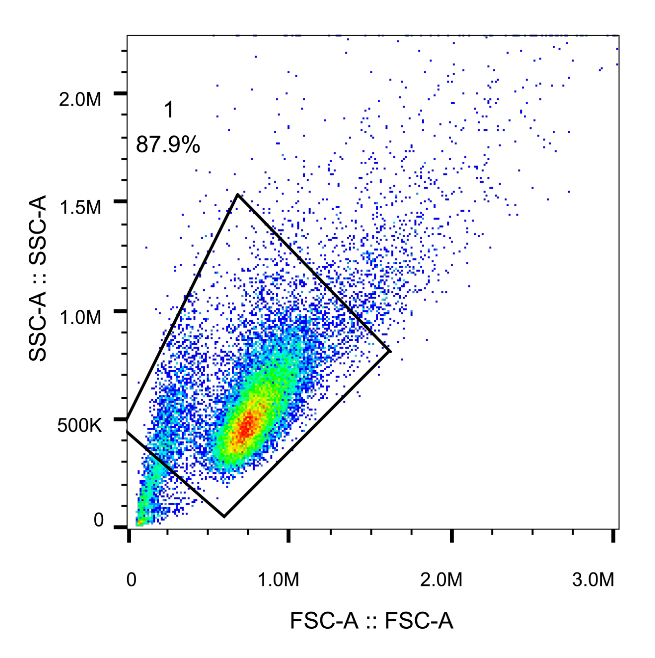


**Figure S2. Initial gating of the main cell population based on FSC-A vs. SSC-A in flow cytometry.** The primary cell population was gated using forward scatter area (FSC-A) and side scatter area (SSC-A) to exclude debris and non-target events. The gated population represented 87.9% of the total events and was used for downstream analysis.


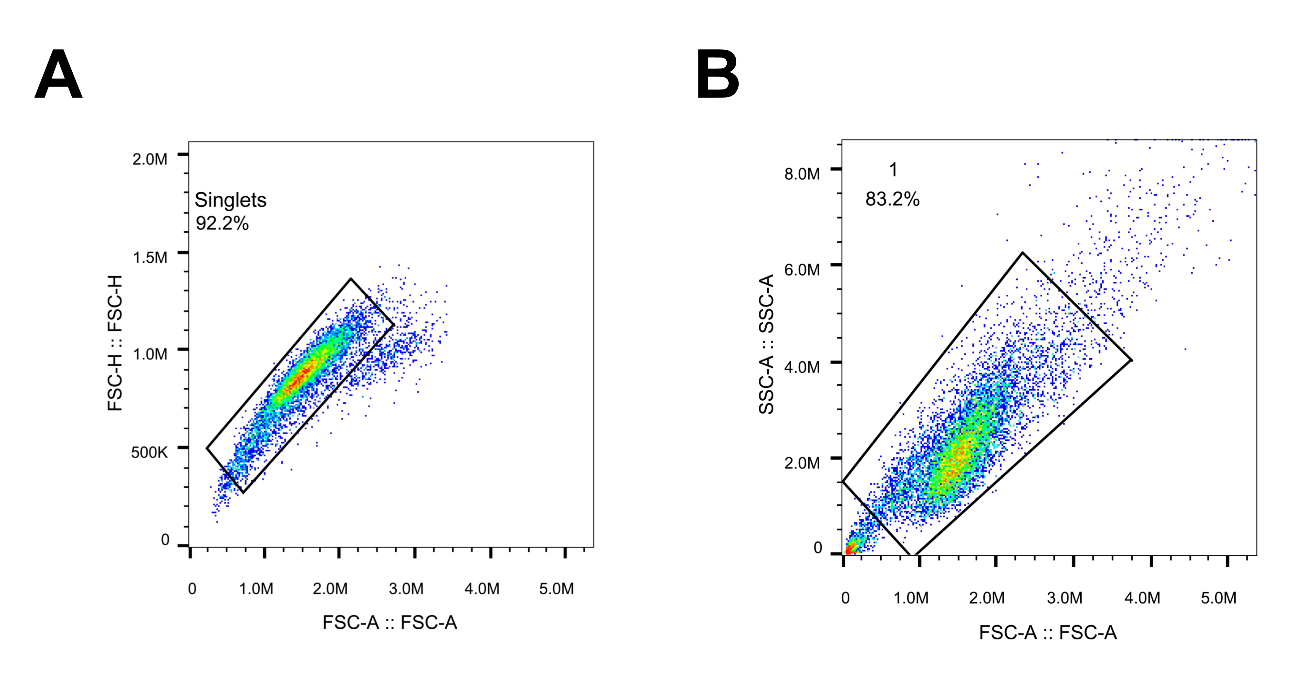
 **Figure S3. Gating strategy for identifying target cell populations by flow cytometry.** (A) Single cells were selected using FSC-A vs. FSC-H to eliminate doublets and aggregates, with singlets comprising 92.2% of events. (B) The main cell population was further gated using FSC-A vs. SSC-A, accounting for 83.2% of total events. This gating strategy was applied for subsequent phenotypic or functional analysis.


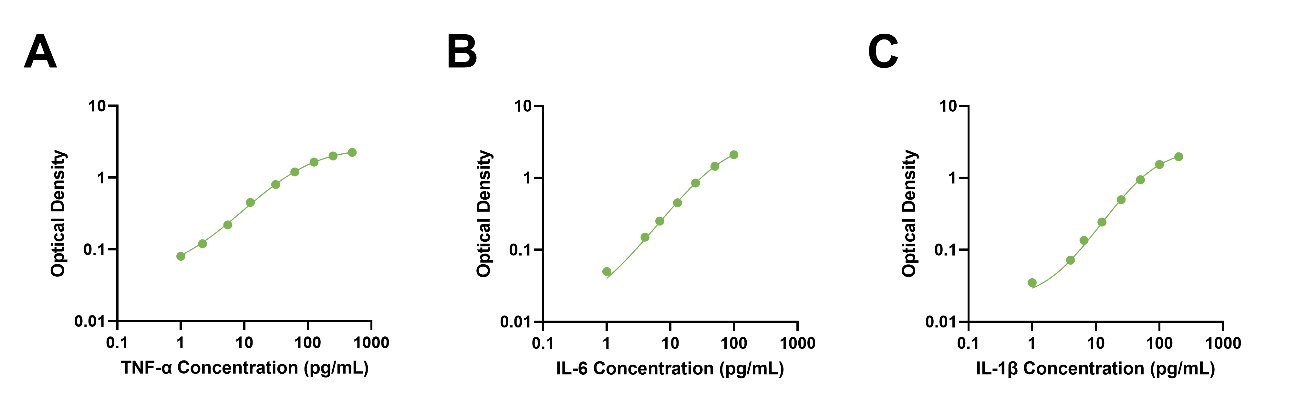


**Figure S4. ELISA standard curves for inflammatory cytokines.** Standard curves for (A) TNF-α, (B) IL-6, and (C) IL-1β, showing the optical density (OD) response across a logarithmic range of cytokine concentrations (0.1–1000 pg/mL). All three cytokines exhibited a clear dose-dependent increase in OD, demonstrating good sensitivity and linearity of the ELISA assay used in this study.
